# Bullying and anxiety/depressive symptoms in Latinx adolescents living with obesity: the mediating role of self-esteem

**DOI:** 10.1007/s12144-024-07259-9

**Published:** 2025-01-09

**Authors:** Padideh Lovan, Devina J. Boga, Alyssa Lozano, Beck Graefe, Shanelle Hodge, Yannine Estrada, Tae Kyoung Lee, Guillermo Prado

**Affiliations:** 1 School of Nursing and Health Studies, University of Miami, Miami, FL USA; 2 Sylvester Comprehensive Cancer Center, Miller School of Medicine, University of Miami, Miami, FL USA; 3 Department of Public Health Sciences, University of Miami Miller School of Medicine, Miami, FL USA; 4 Department of Educational and Psychological Studies, University of Miami, Coral Gables, FL USA; 5 Department of Child Psychology and Education/Social Innovation Convergence, Sungkyunkwan University, Seoul, South Korea

**Keywords:** Bullying, Latinx adolescents, Obesity, Self-esteem, Internalizing symptoms

## Abstract

Bullying is a serious public health issue for adolescents in the United States. Previous studies have demonstrated associations between self-esteem, anxiety/depressive symptoms, and bullying victimization (BV); however, these relationships have not been extensively studied considering the overlap of social identities of Latinx adolescents living with obesity and overweight (LAWO), who are more likely to be victims of bullying. The current study aims to address these gaps by examining the relationship between BV and anxiety/depressive symptoms and the role of self-esteem while considering sex differences among LAWO (*N* = 139; female: *n* = 77, 55.4%; mean age = 12.9 years). Results for overall group showed that BV significantly predicted anxiety/depressive symptoms and self-esteem significantly mediated this relationship. Multigroup mediation analysis resulted in significant mediation by self-esteem for females. Results suggest that interventions that target self-esteem and consider culture would be beneficial for female LAWO.

## Introduction

Bullying is a serious and urgent public health issue among youth and is related to significant ramifications including psychosomatic symptoms, alcohol abuse, drug abuse, self-injuries, and suicide attempts. The consequences of being a victim of bullying during childhood or adolescence may even lead to physical complications during adulthood such as cardiovascular disease (Hertz et al., 2013; Srabstein & Leventhal, 2010). The Centers for Disease Control and Prevention (CDC) has also reported that bullying causes significant damage to the targeted individual, either physically, psychologically, socially, or educationally (CDC, 2021b). Approximately 22.2% of high school students have reported being a target of bullying at school and 16.6% have reported being targeted by cyberbullies (CDC, 2021b; Department of Education, 2021). Therefore, studying the impact of bullying in youth is of a great importance.

### Youth with overweight or obesity are more likely to be victims of bullying

One of the most common reasons for bullying at school is attributed to weight, specifically being overweight and/or obese. Previous studies showed that youth living with overweight and obesity have a higher risk of being bullied (rationally, verbally, and cyber) compared to their non-obese peers, with a higher risk of physical bullies for obese youth (Garnett et al., 2014; Puhl et al., 2011; Waasdorp et al., 2018). In fact, weight-based harassment/bullying is far more common than sexual and socio-economic-related harassment/bullying among youth (Bucchianeri et al., 2013; Koyanagi et al., 2020). There may also be sex differences in weight-based bullying. Research suggests that both boys and girls who are overweight or obese are more likely to be targeted for bullying compared to non-obese youth (Morales et al., 2019). Our study’s population consists of adolescents with overweight/obesity, which provides valuable information regarding the impact of bullying on their mental health since they are more likely to be victims of bullying due to their weight status.

### Bullying can have life-long impacts

Although bullying has several short- and long-term effects on the individual who is bullied, anxiety and depression have been shown to be the most prevalent consequences of being bullied in previous research among youth (Consequences of Bullying Behavior, 2016). Particularly, weight-based bullying leads to several problems among the victimized adolescents including low body satisfaction and self-esteem, as well as depressive symptoms and suicidal thoughts (Eisenberg et al., 2003; Patte et al., 2021). Previous studies have suggested a negative correlation between self-esteem and bullying victimization (Choi & Park, 2018; Jamir et al., 2014; Wu et al., 2021). One particular study found that self-esteem played a mediating role in the relationship between weight status and being bullied (Fox & Farrow, 2009). Furthermore, lower self-esteem is significantly correlated with higher anxiety, depression, and academic stress, which may affect quality of life among adolescents (Nguyen et al., 2019a, b). There are also differences between male and female youth pertaining to self-esteem. It has been found that males have reported higher self-esteem compared to females when they are involved in bullying situations for those in both victim and aggressor roles (Brito & Oliveira, 2013).

### Being bullied varies in different sexes and ethnicities

Being bullied differs across sex and ethnicity. Reports from 2019 among adolescents 12–18 years of age show that a higher percentage of females were bullied compared to males (25.5% vs. 19.1%). Findings of a recent meta-regression analysis that show a decline in face-to-face bullying victimization from the late 1990s to the 2010s, however, that decline has been slower for females and, in fact, there have even been increases in some years (Kennedy, 2021). Further, nearly 25% of Non-Latinx White adolescents have reported experiencing bullying followed by Blacks (22.2%) and Latinx adolescents (18%; (Department of Education, 2021). Although these reports show a higher rate of bullying in non-Latinx Whites, other studies have indicated that Latinx youth who are bullied, are disproportionately impacted by the consequences of bullying, particularly, if they are in a school with fewer Latinx students (Gage et al., 2021). Previous studies have suggested strong correlations between ethnic-based bullying and depression among foreign-born Latinx middle and high schoolers (Cardoso et al., 2018). Another study among Latinx and Black adolescents found that bullying had a mediating effect on the associations between acculturative stress and depression (Forster et al., 2013). A systematic review that examined the correlation between bullying and depression with the inclusion of the Latinx adolescent population affirmed significant associations between bullying and depressive symptoms. Further, Latinx adolescents’ experiences as ethnic minority may have shaped different sociocultural experiences around bullying, compared to their non-Latinx White peers. However, limited data are available on the Latinx population living in the US on bullying and further research is required to understand the relationship between bullying and mental health among Latinx youth (Lutrick et al., 2020).

### Study rationale and objectives

The development of the hypothesized mediating model that explores the role of self-esteem in the association between bullying victimization and anxiety/depressive symptoms during adolescence is grounded in developmental and social psychology frameworks. Adolescence is a critical developmental stage where individuals establish a sense of identity and self-worth, making them particularly vulnerable to external influences such as peer interactions and social feedback (O’Connell et al., 2009). Although studies have examined the impact of bullying on adolescents’ mental health (e.g., anxiety and depression), there are still multiple research gaps. First, little is known about the effects of bullying on Latinx mental health, one of the largest minority populations living in the US (Jones et al., 2021). Second, to the best of our knowledge, no study has addressed sex differences in the associations between bullying and anxiety/depressive symptoms, particularly among Latinx youth with overweight/obesity, who are more likely to be victims of bullying. This study is particularly unique in that it addresses a critical gap in existing research by focusing solely on a population of overweight and obese Hispanic adolescents residing in South Florida. While previous studies have explored the relationship between bullying victimization, self-esteem, and mental health outcomes, few have examined these dynamics within this specific demographic. Given the unique cultural, social, and environmental factors that may influence the experiences of Hispanic youth in the US, especially those who are overweight or obese, this study offers a valuable contribution to the literature by providing insights that are both culturally and contextually relevant. By examining these factors within this underserved group, the study highlights the need for tailored interventions that address the intersecting issues of weight, ethnicity, and mental health in vulnerable adolescent populations.

Therefore, to address these research gaps, our study aims to (1) explore the direct association between bullying victimization and anxiety/depressive symptoms among Latinx adolescents with overweight/obesity; (2) examine the mediating effect of self-esteem between bullying victimization and anxiety/depressive symptoms; and (3) examine the moderating effect of participants’ sex in the mentioned associations among Latinx adolescents with overweight/obesity. We hypothesize that (a) bullying victimization will be negatively associated with self-esteem; (b) self-esteem will be negatively associated with anxiety/depressive symptoms; (c) bullying victimization will be positively associated with anxiety/depressive symptoms; (d) self-esteem will mediate the relationship between bullying victimization and anxiety/depressive symptoms; (e) there will be a moderating effect of sex, where the mediating effect of self-esteem in the relationship between bullying victimization and anxiety/depressive symptoms will be significant in both females and males but the relationship will be stronger in females. Additionally, we utilized baseline data to measure bullying victimization and self-esteem, while anxious and depressive symptoms were assessed six months following the baseline evaluation.

## Methods

### Participants and procedure

Data for the current analysis were from participants in the control condition of a randomized clinical trial that tested a family-based healthy lifestyle intervention which focused on modifying lifestyle behaviors in Latinx adolescents who were overweight and obese. Study details and CONSORT is published elsewhere (Prado et al., 2020). We opted to exclude the intervention arm from our analysis to eliminate the influence of mental health improvements attributed to the intervention, thereby allowing us to observe the impact of the variables in their natural state, unaltered by the intervention’s effects. Data collection was conducted in either English or Spanish, tailored to each participant’s language preference. Eligibility to participate in the study included (1) identifying as a Latinx adolescent in the 7th or 8th grade, (2) living with an adult primary caregiver willing to participate in the study for two years and remaining in South Florida for the study period, (3) adolescent having a Body Mass Index (BMI) ≥ 85th percentile according to their age and sex. Adolescents from 18 middle schools in South Florida were screened using the body silhouette technique to identify their BMI status (Lønnebotn et al., 2018). In efforts to reduce stigma in the recruitment process, adolescents received letters describing the study without explicit BMI criterion and a broad description of the intervention to share with their parents. Parents expressed interest in participation by providing their contact information.

The total sample consisted of 280 participants; the final analytic sample used in this study included 139 participants from the control condition (described in Table 1). The intervention arm was excluded from the analysis because multiple time points of data were utilized, and the effects of the intervention would likely influence the outcomes. Since the intervention specifically aimed to improve the adolescents’ health status, including this group could confound the relationships being examined. As per the inclusion criteria, 100% of the sample identified as Latinx. Approximately 55% of participants identified as female (*n* = 77) and the mean age of the sample was 12.9 years old (*SD* = 0.78). At baseline, around 50% of our sample had a BMI percentile matching that of obese status with the remainder of participants categorized as overweight (CDC, 2021a). The sample included participants from a variety of Spanish speaking countries, about one-third of the parents were born in Cuba, followed by Nicaragua (17.9%), Honduras (10.7%), Colombia (9.3%), Peru (3.6%), and Venezuela (2.9%). Data were collected longitudinally, and baseline and 6-month post baseline were used for this study. This study was approved by the Miami-Dade County Public School System and University of Miami Institutional Review Boards.

**Table 1:** Descriptive statistics of socio-demographic characteristics

Characteristic	*n*/Mean (SD/%)
Adolescents	
Age (years)	12.99 (0.79)
Sex	
Male	62 (44.6)
Female	77 (55.4)
Nativity Status	
US Born	95 (68.3)
Foreign Born	44 (32.7)
Body Mass Index Percentile	93.96 (4.43)
Overweight (85th– 95th percentile)	67 (48.1)
Obese or Severely Obese (> 95th )	72 (51.9)
Parents	
Age (years)	41.70 (6.71)
Sex	
Male	15 (10.7)
Female	125 (89.3)
Nativity Status	
US Born	11 (7.9)
Foreign Born	128 (92.1)
Body Mass Index Percentile	30.23 (5.20)
Normal weight (18.5–24.9)	14 (10.1)
Overweight (25.0–29.9)	61 (43.9)
Obese (≥ 30.0)	60 (43.2)
Household annual income, USD	
<30,000	90 (68.2)
30,000–49,000	26 (18.7)
>50,000	16 (11.5)
Highest education level attained/completed	
No education	1 (0.7)
Elementary	12 (8.6)
High School	57 (41.1)
College	61 (43.9)
Graduate/Professional School	8 (5.8)
Marital Status	
Married	82 (59.0)
Cohabiting	13 (9.4)
Separated	10 (7.2)
Divorced	19 (13.7)
Widowed	2 (1.4)
Never married/not cohabiting	13 (9.4)

### Measures

#### Sociodemographic characteristics

To describe the characteristics of the participants included in the study, demographic measure was administered to capture information on our participants pertaining to sex, nativity status, BMI, age, household income, parents’ education level, and parents’ marital status.

#### Bullying victimization

To capture experiences surrounding bullying, we used two items from the Youth Risk Behavior Surveillance Survey (YRBSS). Bullying was operationalized for the participants in a prompt before the questions, reading as follows: “*Bullying is when 1 or more students tease*,* threaten*,* spread rumors about*,* hit*,* shove*,* or hurt another student over and over again. It is not bullying when 2 students of about the same strength or power argue or fight or tease each other in a friendly way.*” The first item probes whether the participant had ever been bullied on school property and the second item asks about being bullied electronically. Response options for both questions were binary options of yes or no. The Cronbach’s alpha for this scale was α = 0.74 (Underwood et al., 2020).

#### Self-esteem

The Rosenberg Self-Esteem Scale is a unidimensional scale that measures global self-worth by identifying both positive and negative feelings about oneself with 10 items (Rosenberg, 1965). The responses are endorsed on a 4-point Likert-type scale ranging from “*strongly disagree*” to “*strongly agree.*” Sample items include “*I do not have much to be proud of*” and “*I take a positive attitude toward myself.*” Items are reverse scored and summed with higher scores indicating higher self-esteem. The internal consistency for this measure was α = 0.86.

#### Anxiety/depressive symptoms

The Youth Self-Report (YSR) is a comprehensive assessment of anxiety and depression symptoms administered in 11–17-year-old adolescents to assess a variety of behavioral problems and competencies (Achenbach, 1991). There are 8 empirically based mental health related syndromes which includes our outcome for this study: Anxious/Depressed. This syndrome included 13 items (α = 0.85). Sample items include “*I feel that no one loves me*,” “*I am self-conscious or easily embarrassed*,” and “*I am afraid I might think or do something bad.*” Responses are endorsed with a 3-point Likert-type scale where 0 is “*not true*,” 1 is “*somewhat or sometimes true*,” and 2 is “*very true or often true.*” The higher the score, the more anxiety/depressive symptomology present.

### Statistical analysis

**Fig. 1:**
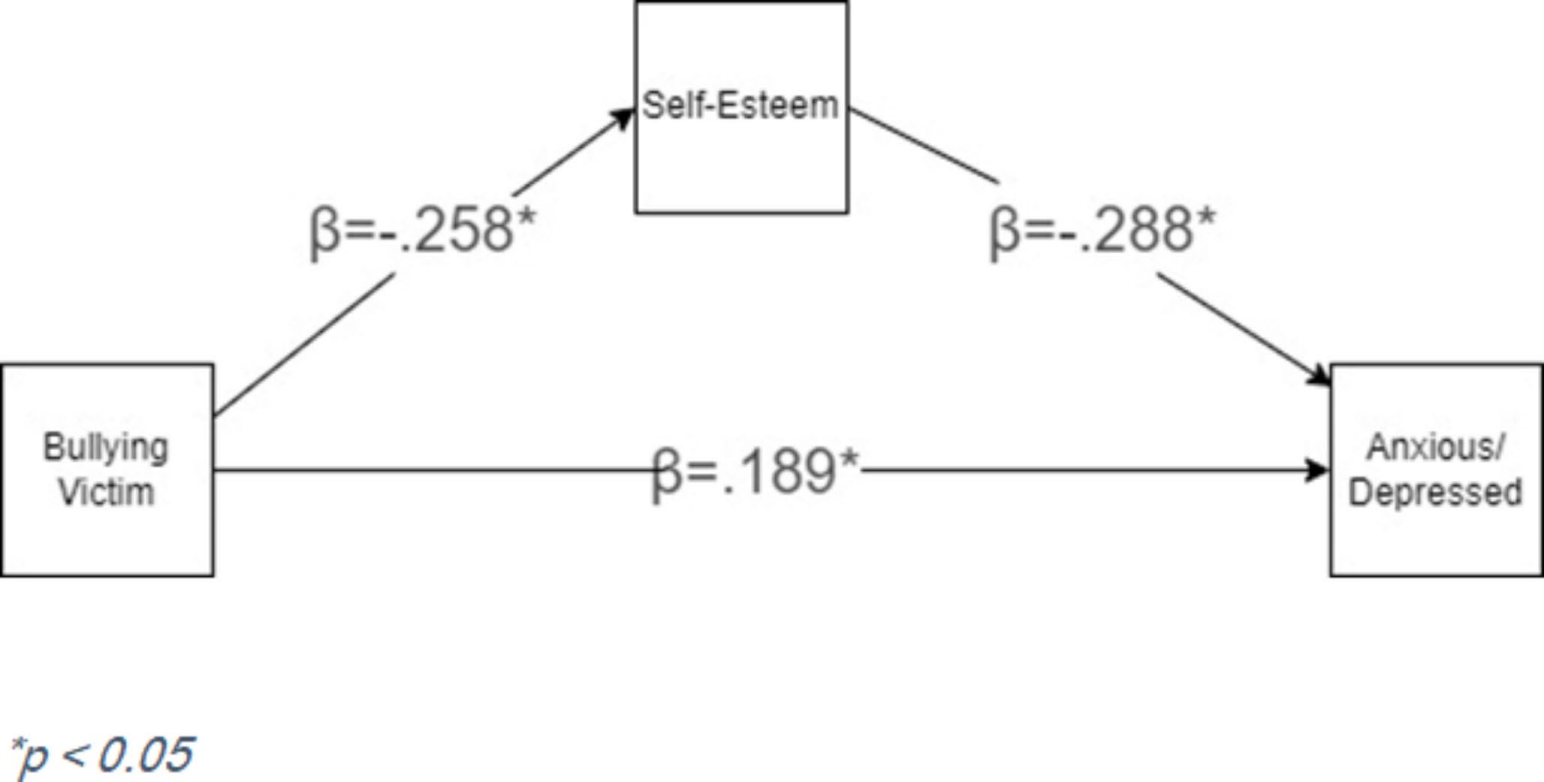
Mediation model with standardized path coefficients

We described the socio-demographic characteristics of our sample using descriptive statistics, means and standard deviations for continuous variables, and frequency and percentage for categorical variables. The current study used bullying victimization as the predictor and self-esteem as the mediator at baseline, and anxious/depressive symptoms as the outcome at 6 months after the baseline assessment. The study model is presented in Fig. 1. We tested the indirect and direct associations of bullying victimization on anxious/depressive symptoms through self-esteem. We also performed a multigroup analysis to test the structural model separately among males and females. Equality constraints across groups were used to compare nested models: (1) a model with certain parameters constrained to be equal and (2) a model with parameters allowed to differ across groups. Model differences between groups for the multigroup model were evaluated based on residual variance. To handle missing data, full-information maximum likelihood (FIML) estimation was used (Enders, 2022). All models were tested under the structural equation modeling framework using Mplus 7.0 (Muthén & Muthén, 2017).

## Results

### Mediation models

The mediation model with standardized regression coefficients for the entire sample is presented in Fig. 1.

Upon examination of the standardized parameter estimates of the model, all paths were found to be significant. The standardized effect of being a bullying victim at baseline significantly predicted anxious/depressive symptoms at 6-months follow-up (β = 0.189, *p* = .039). In analyzing the indirect effects, we found that self-esteem significantly mediated the relationship between being a bullying victim and anxious/depressive symptoms, such that bullying victimization negatively affects self-esteem, which subsequently impacts anxious/depressive symptoms. With every one-point increase in self-esteem score, anxious/depressive symptom scores decreased by 0.288 (*p* = .02). A full description of parameter estimates for the full model is provided in Table 2.

**Table 2:** Regression coefficients: indirect, direct, and total effects

						95% C.I.*		
Model	Type	Effect	$$\:b**$$	SE	$$\:\beta\:***$$	Lower	Upper	$$\:p$$
Overall	Indirect	Bullied - Self-Esteem - Anxious/Depressed	0.929	0.402	0.074	0.025	0.155	0.021
	Component	Bullied - Self-Esteem	−3.023	0.935	−0.258	−0.405	−0.098	0.001
		Self-Esteem - Anxious/Depressed	−0.307	0.093	−0.288	−0.456	−0.100	0.002
	Direct	Bullied - Anxious/Depressed	2.368	1.169	0.189	0.009	0.372	0.039
	Total	Bullied - Anxious/Depressed	3.297	1.144	0.263	0.092	0.442	0.003
Male	Indirect	Bullied - Self-Esteem - Anxious/Depressed	0.637	0.537	0.057	−0.005	0.201	0.244
	Component	Bullied - Self-Esteem	−2.459	1.339	−0.228	−0.449	0.030	0.062
		Self-Esteem - Anxious/Depressed	−0.259	0.145	−0.253	−0.528	0.043	0.081
	Direct	Bullied - Anxious/Depressed	1.493	1.471	0.135	−0.118	0.400	0.306
	Total	Bullied - Anxious/Depressed	2.129	1.460	0.193	−0.061	0.442	0.137
Female	Indirect	Bullied - Self-Esteem - Anxious/Depressed	1.345	0.702	0.095	0.002	0.216	0.099
	Component	Bullied– Self-Esteem	−4.177	1.270	−0.328	−0.473	−0.031	0.002
		Self-Esteem - Anxious/Depressed	−0.322	0.129	−0.291	−0.518	−0.038	0.018
	Direct	Bullied - Anxious/Depressed	3.543	1.993	0.251	−0.126	0.507	0.108
	Total	Bullied - Anxious/Depressed	4.889	1.802	0.346	−0.021	0.553	0.012

*Confidence intervals computed with bootstrapping method

**Unstandardized$$\:\beta\:$$

***Standardized$$\:\beta\:$$

### Multigroup mediation

**Fig. 2:**
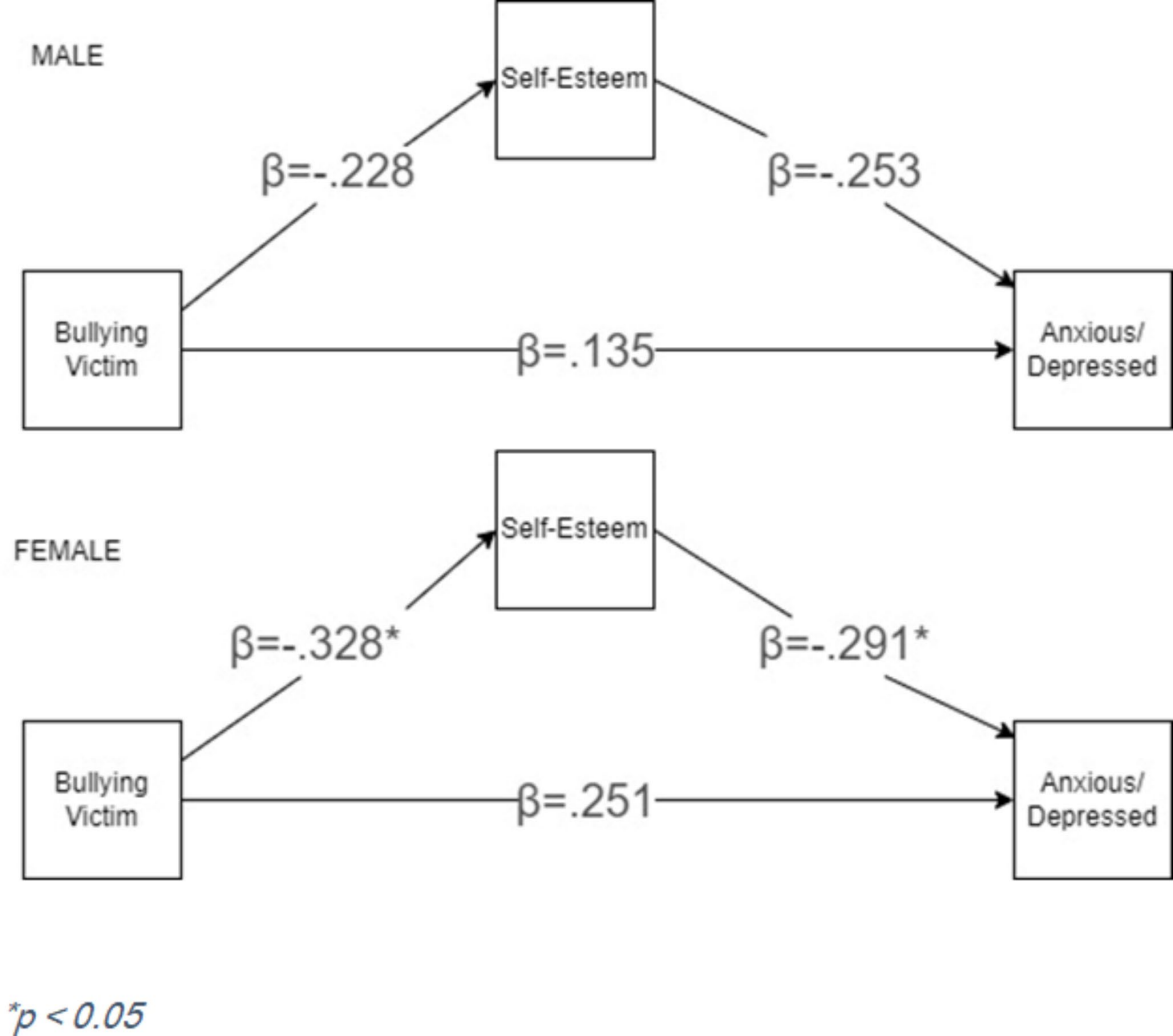
Multigroup mediation model with standardized path coefficients

The multigroup model produced an AIC of 1571.526 and a BIC of 1612.609, indicating that the full overall model performed slightly better than the split model. In addition, model constraint method was used to compare the model invariance between two groups (male and female). Results from this analysis indicate that the male and female models were not significantly different in terms of residual variance (*t* = 1.068, *p* = .286). As shown in Fig. 2, in the model for males, all paths were found to be statistically non-significant. In the model for females, bullying victimization had a significant effect on self-esteem (β = −0.328, *t* = −3.171, *p* = .002), while self-esteem had a significant effect on anxious/depressive symptoms (esteem (β = − 0.291, *t* = −2.356, *p* = .018). The direct effect of bullying victimization on anxious/depressive symptoms did not remain significant for the female model. While sex differences emerged in terms of model coefficient significance, a direct comparison of direct slopes using z-tests between the female and male models indicated that the slopes for each individual path were not significantly different (*p* > .05) between female and male participants. A full description of parameter estimates and significance for each path in the male and female models can be found in Table 2. Additionally, Table 3 presents descriptive characteristics for participants' subgroups, Table 4 presents descriptive statistics for study measures, and Table 5 presents the correlation coefficients for study measures.

**Table 3:** Descriptive statistics of socio-demographic characteristics in adolescent subgroups

Characteristic	*n*/Mean (SD/%)
Male (*n* = 62)	
Age (years)	12.94 (0.72)
Nativity Status	
US Born	47 (74.6)
Foreign Born	16 (25.4)
Body Mass Index Percentile	95.68 (4.05)
Female (*n* = 77)	
Age (years)	13.03 (0.72)
Nativity Status	
US Born	53 (68.8)
Foreign Born	24 (31.2)
Body Mass Index Percentile	92.52 (4.23)

**Table 4:** Descriptive statistics for study measures

	Overall		Male		Female	
Measure	Mean/Frequency	SD/%	Mean/Frequency	SD/%	Mean/Frequency	SD/%
Anxiety/Depressive Symptoms	5.37	4.46	4.49	3.92	6.07	4.76
Self Esteem	20.56	4.96	21.21	4.94	20.05	4.95
Bullied	32	22.9	18	28.6	14	18.2

**Table 5:** Correlation coefficients for study measures

	Anxiety/Depressive Symptoms	Self Esteem
Overall		
Anxiety/Depressive Symptoms	1	
Self Esteem	−0.46*	1
Bullied	0.36*	−0.26*
Male		
Anxiety/Depressive Symptoms	1	
Self Esteem	−0.25	1
Bullied	0.41*	−0.23
Female		
Anxiety/Depressive Symptoms	1	
Self Esteem	−0.57*	1
Bullied	0.40*	−0.33*

*p < .01

## Discussion

Findings of this study partially support this study’s hypotheses. We found that (a) bullying victimization was negatively associated with self-esteem; (b) self-esteem was negatively associated with anxiety/depressive symptoms; (c) bullying victimization was positively associated with anxiety/depressive symptoms; (d) self-esteem mediated the relationship between bullying victimization and anxiety/depressive symptoms; (e) self-esteem mediated the relationship between bullying victimization and anxiety/depressive symptoms for females, but this relationship was not significant for males.

Child mental health has been assessed in terms of internalizing and externalizing behaviors (Eisenberg et al., 2001). Anxiety and depression are examples of internalizing behaviors, which often result from bullying and other external stimuli. Internalizing behaviors displayed by children are directed inward and often indicate a child’s psychological and emotional state. An individual exhibiting internalizing behavior may mentally withdraw from the world around them, directing their feelings inwardly, therefore their emotions are not visible to the surrounding world (Eisenberg et al., 2001). Further, internalizing behaviors often may lead to externalizing behaviors, which habitually violate social norms or standards such as lashing out, physical assault, defiance and substance abuse (Eisenberg et al., 2001) (Daughters et al., 2009; Lau et al., 2021; Martel, 2013; Maschi et al., 2008). Therefore, it is essential to understand the factors associated with internalizing behaviors.

Our study focused on a distinct group of Latinx adolescents who are overweight or obese. Previous research has identified obesity as a risk factor for bullying, highlighting that both obesity and bullying can adversely affect the mental health of children. It was also noted that adolescents with obesity who experience bullying tend to have elevated depression and anxiety scores (Şahin & Kırlı, 2021). Although our research did not evaluate the effects of bullying across varying weight statuses, our findings indicated the significant link between bullying and the presence of anxiety/depressive symptoms in adolescents with overweight or obesity, mirroring the conclusions of the aforementioned study. Moreover, our study results align with previous findings, demonstrating significant correlations between increased bullying and decreased self-esteem, as well as associations between lower self-esteem and heightened levels of anxiety and depression among adolescents (Ayoub et al., 2021; Dou et al., 2022; Nguyen et al., 2019a, b). It has also been suggested that self-esteem may serve a protective role against anxiety and depression in adolescents (Henriksen et al., 2017). Further, evidence suggests that self-compassion acts as a mediator in the relationship between bullying and anxiety/depression (Yan et al., 2022). However, to the best of our knowledge this is the first study that found a mediating effect of self-esteem between bullying and anxiety/depressive symptoms is Latinx youth with overweight/obesity.

Identifying as a bully-victim, bully and/or victim of bullying is associated with various mental health consequences, including low self-esteem, increased stress and anxiety, depressive symptoms, and suicidal ideation (Brunstein Klomek et al., 2007; Holt et al., 2015; O’Moore & Kirkham, 2001). Literature pertaining to adolescent bullying by gender suggests that female adolescents not only experience bullying more often than males, but they are also more likely to exhibit internalizing behaviors, compared to males’ preponderant display of externalizing behaviors (Liu et al., 2011; Silva et al., 2013). Female’s tendency to internalize their emotions, and male’s tendency to externalize behaviors ultimately contributes to gender differences in the wide-ranging mental and physical effects of bullying. Children that display externalizing behavior often suppress auxiliary emotions internally. Researchers have found it useful to examine internalizing behaviors independently from their supplemental externalizing behaviors to better understand the complexity of bullying effects (Liu et al., 2011). While this study did not examine externalizing issues, prospective research studies should evaluate externalizing issues with regard to gender and the mediating effects of self-esteem in Latinx children with obesity.

Our findings indicated significant associations between bullying, self-esteem, and anxiety/depressive symptoms in females but not in males, which may be due to the facts that females in general experience bullying more than males at school. Reasons for the gender differences in involvement in bullying may be attributed to the traditional roles played among males and females, either as victims or perpetrators (Silva et al., 2013). For example, bullying among males is often physical, and easily addressed due to its visibility, whereas females often engage in bullying activities or relational aggression that is less visible and easily concealable such as spreading rumors via social media and/or exclusion from social groups (Crick & Grotpeter, 1995; Olweus et al., 2019; Silva et al., 2013; Underwood, 2003). Additionally, female adolescents are significantly more likely to be bullying perpetrators–victims than their male peers (Rice et al., 2015) meaning that they are not only the prevailing victims of bullying, but they are also the primary perpetrators (i.e., bully-victims). The heightened incidence of bullying among females is related to varying perceptions of bullying between males and females. A study conducted by Gordillo (2012) puts these differences in perspective, study results showed that most male adolescents interpreted various forms of bullying (i.e., teasing, name-calling, or making rude gestures) as mechanisms of interaction between peers. When presented with the same scenarios, female adolescents attributed the same behaviors as intentional harm resulting from an imbalance of power.

For Latinx females, the increased likelihood of internalizing symptoms (e.g., depression and anxiety) may also be attributed to acculturation (Lorenzo-Blanco et al., 2011, 2012). The process of acculturation, where individuals adapt to a culture that is different from their heritage culture (Schwartz et al., 2010), significantly impacts Latinx families and may lead to family conflict and reduced family cohesion. Latinx females may in turn have a heightened sensitivity to this conflict and/or lack of cohesion that may consequently lead to higher levels of depressive symptoms relative to Latinx males (Lorenzo-Blanco et al., 2011; Zayas et al., 2005). For Latinx adolescents (and females in particular) with overweight and/or obesity, this construct may impact dietary and activity-related behaviors because obligation to family may supersede attention to oneself as it relates to diet and physical activity (Larios et al., 2009; McLaughlin et al., 2017). For example, Latinx parents may encourage and/or pressure their children to eat due to cultural beliefs that heavier children reflect good parenting and health, and adolescents may not oppose these instructions in order to maintain family harmony (Rosas et al., 2010). These controlling behaviors are associated with an increased risk for unhealthy eating among Latina adolescents, leading to increased BMI/weight that may lead to weight-based bullying (Arredondo et al., 2006; Power et al., 2015).

Multiple theoretical frameworks exist to elucidate the underlying rationale for bullying. For instance, a particular study identified three core attributes of bullying: goal-directed behavior, an imbalance of power, and infliction of harm. This perspective offers a more nuanced understanding of bullying, extending beyond mere aggression to examine its deeper complexities (Volk et al., 2014). The Theory of Planned Behavior has been proposed to explain bullying behavior among school students, offering a detailed analysis of how individual attitudes, societal norms, and perceived behavioral control contribute to the formation of bullying behaviors (Azjen & Fishbein, 1988; Rigby, 2022). Furthermore, the Comprehensive Bullying Model expands on this by not only examining the factors influencing bullying but also describing the outcomes following the formation of an intention to bully. This model integrates a broader spectrum of influences and consequences, providing a more in-depth understanding of the dynamics of bullying (Rigby, 2021, 2022). Previous research has highlighted various theoretical frameworks related to bullying, unanimously indicating the harmful impacts of bullying on the victim’s mental health and that bullying is shaped by multidimensional factors spanning from individual and family environment to the social and school environments. Consequently, addressing bullying is crucial for individuals mental health and quality of life and effectively necessitates multifaceted strategies that encompass these diverse influences (Clark et al., 2020; Espelage & Swearer, 2009).

### Limitations and future directions

The current study has its limitations. First, the measures used in the study were all self-report, and thus they are subject to respondent bias. There is a risk of social desirability bias when broaching the topic of bullying with adolescents that could lead to underreporting or even over reporting if aggression is rewarded with the attention of peers (Branson & Cornell, 2009). Second, the generalizability of our sample is limited as the sample consisted of Latinx adolescents residing in South Florida who were overweight or obese, the relationship between bullying victimization, self-esteem and anxiety/depressive symptoms may be reflected differently in other cultures and/or groups. The questions related to bullying victimization did not include more detail on perceptions of cause of bullying to parse out weight-based bullying vs. other types of bullying. Lastly, only using the intervention arm and subgrouping by gender in the sample lead to a reduction in statistical power. Even though based on the number of participants, our study power is considered medium, studies related to human behaviors are generally known to have low power and it is possible that our significant findings may be due to the unique characteristics of our study population (Latinx youth with overweight/obesity), which may lead to clearer, more pronounced effects making it easier to find significant results (Dumas-Mallet et al., 2017; Greenland et al., 2016). Therefore, subsequent studies should investigate gender subgrouping with larger samples to increase generalizability. Future research directions include using mixed methods to understand differences in how Latinx adolescents experience and recognize bullying to better understand useful strategies for intervention and prevention. It is possible that adolescents may benefit from interventions that are multicomponent in targeting nutrition and mental health with multiple levels through schools, parents and peers.

### Implications for practice and prevention

Taken together, tackling bullying among youth is essential for enhancing mental health (e.g., anxiety and depression). Given that bullying stems from a variety of causes, ranging from individual factors to family and social environments, successful interventions for bullying prevention must employ comprehensive strategies that address these multifarious influences. Anti-bully school-policies have been found to be successful strategies to decrease bullying among youth at school and provide a healthier and safer environment for the students (Hall, 2017). Furthermore, prior research underscores the considerable effect of parenting interventions in mitigating bullying, offering substantial evidence to improve policies and practices. This research advocates for actively facilitating parental engagement in reducing bullying behavior, augmenting communication between parents and children concerning bullying, and enhancing parenting skills (Chen et al., 2021).

For Latinx adolescents with overweight and/or obesity, there are several factors that heighten the risk for the negative effects of bullying victimization on self-esteem and consequently anxiety and depression (Hepburn et al., 2012; Klomek et al., 2013). This highlights the need for family-based interventions to help address the multiple levels of influence that may impact Latinx adolescents’ risk factors such as self-esteem, bullying, and bullying related outcomes like anxiety and depression (Koehly & Loscalzo, 2009). Family-based interventions are among the most efficacious/effective preventive interventions (Austin et al., 2005) and for Latinx adolescents, have shown crossover effects on multiple outcomes (Fernandez et al., 2021; Perrino et al., 2016; Vidot et al., 2016). Further, given the role of “*familismo*” and commitment to family among Latinx groups, it follows that families (e.g., parent and adolescent) should both be engaged in prevention efforts (Cuy Castellanos et al.). In fact, parent support and parent-child relationship quality have both been highlighted as protective factors against bullying (Lereya et al., 2013; Lin et al., 2018; Wang et al., 2010). Parents can help to promote healthy psychosocial adjustment in adolescents to buffer the negative effects of bullying on adolescent mental health.

Although family is salient for Latinx individuals, the context of self-esteem and bullying may lend itself to an intrapersonal focus for adolescents to effectively process and work through challenges related to bullying. It may be that in addition to conjoint learning processes between parents and adolescents, adolescents may also need skills related to developing effective coping strategies that can help them address bullying and also target their self-esteem (Himmelstein & Puhl, 2019; Rosenthal et al., 2015). This may be effective through adolescent-only groups that bring youth with shared experiences together for social support and for a conjoint skill learning process. Additionally, while the face-to-face bullying has trended downwards, studies have found that there have been increases in cyberbullying (Kennedy, 2021; Rigby & Smith, 2011). The increase in cyberbullying is not surprising given the context of the COVID-19 pandemic (Barlett et al., 2021). Given the possible differential impact of cyberbullying and the differences seen in this study between males and females, it will be imperative that preventive interventions offer a tailored approach based on the unique needs and experiences of males and females (Lee et al., 2021).

## Conclusion

Findings from this study demonstrated significant associations between bullying, self-esteem, and anxiety/depressive symptoms in Latinx adolescents with overweight or obesity. Additionally, we found that self-esteem plays a mediating role in the relationship between bullying and anxiety/depressive symptoms. In our comparison of this relationship between male and female adolescents, we discovered that it was only significant among Latinx females, with no significant correlation observed in males. Future research should use a larger sample size to test these relationships. Future interventions are likely to achieve greater success by adopting a multi-level approach, ranging from the creation of anti-bullying school policies and family involvement to personal interventions aimed at boosting self-esteem and discouraging bullying behavior among adolescents.

## Data Availability

Data is not publicly available to protect study participant privacy.
